# Comparison of Odorants in Beef and Chicken Broth—Focus on Thiazoles and Thiazolines

**DOI:** 10.3390/molecules27196712

**Published:** 2022-10-09

**Authors:** Huiqi Yeo, Dimitrios P. Balagiannis, Jean H. Koek, Jane K. Parker

**Affiliations:** 1 Department of Food and Nutritional Sciences, University of Reading, Whiteknights, Reading RG6 6DZ, UK; 2 Foods Innovation Centre Unilever, Bronland 14, 6708 WH Wageningen, The Netherlands

**Keywords:** beef, chicken, GC-O, thiazole, thiazoline

## Abstract

The shift in consumer landscape towards vegan, vegetarian and flexitarian diets has created an unprecedented challenge in creating meat aroma from plant-based alternatives. The search for potential vegan solutions has thus led to a renewed interest in authentic meat flavour profiles. To gain a better understanding of the qualitative odour differences between boiled beef and boiled chicken, aroma extracts were isolated using Likens-Nickerson simultaneous distillation-extraction (SDE), selected expressly because the in-situ heating of the sample facilitates the capture of aroma intermediates during the cooking process, thereby mimicking the cooking of meat in stocks and stews. The extracts were then analysed by Gas Chromatography-Mass Spectrometry (GC-MS) and GC-Olfactometry (GC-O). Most of the volatiles identified in this study were sulfur-containing compounds, such as sulfides, thiols, mercaptoaldehydes and mercaptoketones, which are derived from the Maillard reaction. Meanwhile, lipid oxidation results in the formation of unsaturated aldehydes, such as alkenals and alkadienals. Families of thiazoles and 3-thiazolines were found in the extracts. Two novel 3-thiazolines (5-ethyl-2,4-dimethyl-3-thiazoline and 2-ethyl-4,5-dimethyl-3-thiazoline) which may also contribute to the meaty aroma were identified in this work and synthesised from their respective aldehyde and mercaptoketone precursors.

## 1. Introduction

The rapid growth in vegan, vegetarian and flexitarian diets has led to renewed interest in the development of process flavourings with authentic flavour profiles of cooked meat, which is an important determinant of consumer acceptance [1]. Despite the advances in analytical techniques, recent studies were more interested in specific animal breeds [2,3,4,5], distinct animal parts [5,6] or special cooking techniques [7,8,9] rather than a comparison between species using the same cooking and extraction technique.

The characteristic aroma of cooked meat is generated from thermally induced reactions, principally the Maillard reaction and lipid oxidation, between non-volatile components of lean and adipose tissues during heating. These complex reaction pathways lead to the formation of a diverse range of products, accounting for the large number of volatile compounds found in cooked meat [2,3,4,5,6,7,8,9]. Aroma compounds produced during the Maillard reaction are typically responsible for the savoury, meaty (e.g., S-containing compounds such as 2-methyl-3-furanthiol), roast (e.g., pyrazines such as 2-ethyl-3,5/6-dimethylpyrazine) and boiled characters. Meanwhile, those derived from lipid oxidation (e.g., saturated and unsaturated aldehydes such as 2-alkenals and 2,4-alkadienals) impart fatty aromas to cooked meat and can contribute to species-specific notes [10].

In this study, the volatiles present in boiled chicken (BC) and boiled beef (BB) were isolated using Likens-Nickerson simultaneous distillation-extraction (LN–SDE), a technique which offers the advantage of in situ heating and extraction. This mimics the cooking of meat in stocks and stews in a kitchen setting and avoids the loss of aroma during sample transfer inevitable in other methods. Equipped with more sensitive analytical techniques as compared to decades ago, the aim of this work is to gain a better understanding of the qualitative odour differences (i.e., odour activity, quality and intensity) between these species based on a fair comparison (i.e., same cooking and extraction technique), which will be useful in the creation and modification of process flavourings in products such as soups, stocks, and bouillons.

## 2. Results and Discussion

### 2.1. Similarities between Boiled Beef (BB) and Boiled Chicken (BC)

The aroma extracts of both BB and BC had an intense aroma characteristic of their respective species and the volatiles identified by GC-O and GC-MS for BB and BC extracts are listed in Table 1. A total of 58 and 64 odour-active volatiles were identified in BB and BC, respectively. The major classes of compounds in both extracts comprised S- and/or N-containing compounds, including heterocycles such as thiazoles, thiazolines and pyrazines, as well as straight chain saturated and unsaturated aldehydes, alcohols and ketones.

Many of the early-eluting odorants (LRI < 500 on DB-5 column) were highly volatile and potent S-containing compounds, namely hydrogen sulfide, methanethiol, ethanethiol and 2-propanethiol. Thus, some were not detected by GC-MS and only by GC-O as the human nose can be a more sensitive detector. Nevertheless, the majority of the compounds with the highest total odour intensity scores and detection frequencies in both the extracts were S-containing compounds, such as 2-methyl-3-furanthiol (cooked meat, roasted), 2-furylmethanethiol (roasted, burnt) and 3-(methylthio)propanal (potato), which were among the primary odorants identified in chicken broth [11]. As observed for these compounds, not all S-containing compounds impart meaty odours. Meanwhile, the N-containing compounds, tetramethylpyrazine and 2-ethyl-3,5-dimethylpyrazine, imparted carboard, earthy and medicinal notes. As for the N- and S-containing heterocycles, 3-thiazolines and thiazoles, odours ranging from savoury (meaty, fatty and onion) to sweet (popcorn, nutty and roasted rice) were detected and these will be discussed in greater detail in Section 2.3. The Strecker degradation products also contributed to the aroma of both the extracts: 3-methylbutanal (malty, cocoa, nutty), 2-methylbutanal (chocolate liquor, aldehydic) and phenylacetaldehyde (floral, rose, perfume, green) are derived from leucine, isoleucine [12] and phenylalanine [13], respectively.

Besides the Strecker aldehydes, carbonyl compounds derived from the oxidative degradation of unsaturated fatty acids were also identified in both extracts. (*E*,*E*)-2,4-Decadienal (fatty, fried), (*E*)-2-nonenal (fatty, aldehydic, waxy), 1-octen-3-one (cooked mushroom) and (*E*,*E*)-2,4-nonadienal (fatty) are formed from the autoxidation of n-6 fatty acids such as linoleate [14] and arachidonate [15,16]. Meanwhile, aldehydes such as octanal (fruity, sweet) and nonanal (fatty, citral, leafy) originate from the autoxidation of n-9 fatty acids such as oleate. Although none of these carbonyl compounds identified possessed aroma characteristics resembling the complete spectrum of cooked chicken [17], it was reported that the removal of carbonyl compounds from the volatile fraction resulted in a loss of chicken odour and an intensification of meaty odour [18]. However, it was also demonstrated that the omission of (*E*,*E*)-2,4-decadienal alone did not result in a significant aroma change [19]. Thus, it is likely that it is the delicate balance of the group of carbonyl compounds which contribute to the overall aroma rather than a single aroma compound alone.

**Table 1:** Odour-active compounds in the boiled beef (BB) and boiled chicken (BC) extracts.

Compound	Description ^a^	BB						BC						ID ^f^	Ref ^g^
		Odour		LRI_DB-5_^d^		LRI_ZB-Wax_^e^		Odour		LRI_DB-5_^d^		LRI_ZB-Wax_^e^			
		Int ^b^	Det freq ^c^	GC-O	GC-MS	GC-O	GC-MS	Int ^b^	Det freq ^c^	GC-O	GC-MS	GC-O	GC-MS		
Hydrogen sulfide	Faecal, rotten egg, sulfur	31	6	<500	<500	<800	n.d.	28	6	<500	<500	<800	n.d.	O, ms, lri	[20,21]
Acetaldehyde	Sweet solvent	9	3	<500	<500	<800	n.d.	15	3	<500	<500	<800	695	O, MS, LRI	
Methanethiol	Leftover chicken, faecal, rotten cabbage	35	6	<500	<500	<800	n.d.	24	5	<500	<500	<800	n.d.	O, ms, lri	[22,23]
Ethanethiol	Town gas, sulfur	18	4	<600	n.d.	<800	n.d.	12	2	<500	n.d.	<800	n.d.	O, lri	[22,24]
2-Propanethiol	Raw onion	27	4	<600	n.d.	n.d.	n.d.	43	6	<500	<500	<800	n.d.	O, lri	[24]
Formic acid	Mustard, brassica,	36	5	<500	n.d.	n.d.	n.d.	n.d.	n.d.	n.d.	n.d.	n.d.	n.d.	O, MS, LRI	
2,3-Butanedione	Buttery, cheesy, sweaty, creamy	35	6	574	n.d.	987	987	21	5	586	n.d	985	980	O, MS, LRI	
1-Propanethiol	Raw onion, musty, meaty	13	4	603	608	n.d.	n.d.	19	6	603	608	n.d.	845	O, MS, LRI	
3-Methylbutanal	Malty, cocoa, nutty	36	6	646	644	921	919	29	6	644	646	925	931	O, MS, LRI	
2-Methylbutanal	Chocolate liquor, aldehydic	7	3	655	654	914	915	13	3	652	656	915	925	O, MS, LRI	
Unknown	Onion, soil	-	-	-	-	-	-	10	3	654	-	-	-	-	
2,3-Pentanedione	Creamy, buttery, cheesy, sweaty	31	6	696	696	1061	1068	16	5	695	697	1058	1080	O, MS, LRI	
Unknown	Biscuit, pastry, onion, cat’s pee,	-	-	-	-	-	-	16	4	735	-	-	-	-	
Methanedithiol	Raw/rotting onion, catty, petroleum	38	6	737	n.d.	n.d.	n.d.	n.d.	n.d.	n.d.	n.d.	n.d.	n.d.	O, lri	[25]
Dimethyl disulfide	Raw/rotting onion, sulfur, bad egg	23	6	745	744	1080	1072	18	4	744	744	1081	1083	O, MS, LRI	
1-(Methylthio)propane	Onion, pungent, paint, petrol	36	6	766	763	n.d.	n.d.	43	6	765	764	1256	1259	O, MS	
Hexanal	Green, fatty, grassy	36	6	799	801	1097	1082	32	6	799	801	1070	1101	O, MS, LRI	
Mercaptopropanone	Vegetable, sulfur, garlic, beer headspace	28	6	805	n.d	1352	n.d.	22	4	805	n.d	1353	1351	O, lri	[26,27]
Unknown	Brothy, meaty	10	2	813	-	-	-	n.d.	n.d.	n.d.	n.d.	n.d.	n.d.	-	
3-Mercapto-2- butanone	Vegetable, sulfur, pyrazine, cardboard	8	2	817	818	1270	n.d.	17	4	816	818	1265	n.d	O, MS, LRI	
3-Methyl-2-butene-1-thiol	Stewed onion, garlic, meaty, beer headspace	26	6	823	n.d	n.d.	n.d.	26	5	823	n.d	n.d	n.d	O, LRI	
1-(Methylthio)- ethanethiol + Ethyl methyl disulfide	Rotting onions, sulfur, catty, petroleum	38	6	845	847	1221	n.d.	40	6	846	847	1222	1231	O, MS, LRI	
				845	848	1142	1134			846	848	1148	n.d.	O, ms, lri	[25,28]
2-Methyl-3-furanthiol	Roasted, porridge oats, sulfur, faecal	41	6	866	872	1303	n.d.	32	5	869	874	1330	1320	O, MS, LRI	
Trimethyloxazole	Popcorn, nutty, vegetable	20	5	869	851	1150	n.d.	n.d.	n.d.	n.d.	n.d.	n.d.	n.d.	O, MS, LRI	
2-Methyl-2-thiazoline	Boiled/rotting onion, catty, sulfur, medicinal	22	5	876	873	1275	n.d.	45	6	877	884	1278	1284	O, MS, LRI	
2,4-Dimethylthiazole	Meaty, brassica	11	3	890	889	1270	1283	14	4	883	889	1260	1276	O, MS, LRI	
(*Z*)-4-Heptenal	Lamb fat, potato, meaty, buttery	49	6	895	900	1234	n.d.	38	6	898	900	1240	n.d.	O, MS, LRI	
Heptanal	Fruity	n.d.	n.d.	n.d.	n.d.	n.d.	n.d.	5	1	899	903	1183	1189	O, MS, LRI	
3-Mercapto-2-pentanone	Onion, catty	20	4	898	903	1346	n.d.	25	4	900	903	1353	1358	O, MS, LRI	
3-(Methylthio)propanal	Potato	46	6	901	909	1451	1467	33	5	905	909	1443	1448	O, MS, LRI	
2-Furylmethanethiol + 2-Mercapto-3-pentanone	Stewed potato, meaty, beef, gravy	39	5	905	912	1430	1432	34	5	909	912	1438	1431	O, MS, LRI	
				905	n.d.	n.d.	n.d.			909	n.d	n.d.	n.d.	O, lri	[29]
2,4-Dimethyl-3-thiazoline	Meaty, brothy	n.d.	n.d.	n.d.	n.d.	n.d.	n.d.	24	5	928	928	n.d.	n.d.	O, MS, LRI_S_	
2-Acetyl-1-pyrroline	Basmati rice, pandan	42	6	918	924	1322	1327	39	6	920	924	1321	1323	O, MS, LRI	
Propyl 2-methyl-butanoate	Perfume, fruity, saffron	11	3	947	944	n.d.	n.d.	n.d.	n.d.	n.d.	n.d.	n.d.	n.d.	O, ms, lri	[30]
2,4-Dimethyl-5ethyl-oxazole	Roasted/grilled meat	n.d.	n.d.	n.d.	n.d.	n.d.	n.d.	24	5	928	928	n.d.	n.d.	O, ms, lri	[31]
4-Mercapto-4-methyl-2-pentanone	Boiled chicken	n.d.	n.d.	n.d.	n.d.	n.d.	n.d.	7	2	938	936	1385	n.d.	O, ms, lri	[21,32]
(*E*)-2-Heptenal	Green, citral, waxy	17	4	961	961	1318	1311	n.d.	n.d.	n.d.	n.d.	n.d.	n.d.	O, MS, LRI	
Unknown	Fruity, cat’s pee, blackcurrant	9	2	963	-	-	-	-	-	-	-	-	-	-	
1-Heptanol	Mushroom, fusel	n.d.	n.d.	n.d.	n.d.	n.d.	n.d.	17	3	973	973	1455	1459	O, MS, LRI	
Dimethyl trisulfide	Meaty, (pickled) onion	11	2	967	976	1362	1360	9	2	977	976	1352	1351	O, MS, LRI	
1-Octen-3-one + 1-Octen-3-ol	Raw mushroom, green, flower	39	6	976	982	1291	1284	13	2	976	983	1298	1295	O, MS, LRI	
				976	982	1449	1451	13	2	976	983	1455	1457	O, MS, LRI	
(*Z*)-1,5-Octadien-3-one	Geranium	21	4	978	n.d	n.d	n.d.	19	4	981	n.d.	1361	n.d.	O, lri	[33]
2-Methyl-3-octanone	Vegetable, earth, plastic, garlic	19	3	987	986	n.d.	n.d.	n.d.	n.d.	n.d.	n.d.	n.d.	n.d.	O, ms, lri	[34]
Trimethyl-3-thiazoline (I)	Pickled onion, cat’s pee	31	5	990	990	1367	1363	17	3	990	990	1364	1351	O, MS, LRI_S_	
Trimethyl-3-thiazoline (II)	Meaty, fried onion	16	3	994	992	1373	1380	27	5	993	999	1376	1367	O, MS, LRI_S_	
Octanal	Fruity, candy	25	4	999	1005	1279	1265	11	2	1003	1005	1286	1288	O, MS, LRI	
Trimethylisothiazole	Odd sulfur, cardboard, earthy, green	32	6	1010	1008	1395	n.d.	n.d.	n.d.	n.d.	n.d.	n.d.	n.d.	ms, lri	[35]
(*E*,*E*)-2,4-Heptadienal	Earthy, fatty, savoury	n.d.	n.d.	n.d.	n.d.	n.d.	n.d.	5	2	1016	1016	1477	1482	O, MS, LRI	
2-Acetylthiazole	Toasted, biscuit, basmati rice	20	5	1021	1024	1648	1657	24	5	1020	1023	n.d.	1637	O, MS, LRI	
3-Octen-2-one	Earthy, plastic	11	3	1039	1041	1376	n.d.	n.d.	n.d.	n.d.	n.d.	n.d.	n.d.	O, MS, LRI	
4-Ethyl-2-methyl-thiazole	Popcorn, basmati, barley, biscuit, roasting skin	n.d.	n.d.	n.d.	n.d.	n.d.	n.d.	16	3	1047	1046	1429	1427	O, MS, LRI_S_	
Phenylacetaldehyde	Floral, rose, green, perfume	26	6	1047	1052	1644	1656	20	4	1051	1052	1650	1636	O, MS, LRI	
2-Acetyl-1,4,5,6-tetrahydropyridine	Popcorn, roasted skin, fried rice, bread	13	3	1056	1053	n.d.	n.d.	17	3	1053	1053	n.d.	n.d.	O, ms, lri	[36]
Trimethyl-5-hydroxy-3-thiazoline	Roasted, fatty chicken skin	n.d.	n.d.	n.d.	n.d.	n.d.	n.d.	14	2	1055	1060	n.d.	n.d.	ms	
2-Acetylpyrrole	Rice, popcorn, toasted	n.d.	n.d.	n.d.	n.d.	n.d.	n.d.	17	3	1069	n.d.	1969	1970	O, MS, LRI	
2-Ethyl-3,5-dimethyl-pyrazine	Cardboard, pyrazine, cocoa, soil	24	5	1075	1076	n.d.	n.d.	19	4	1078	1081	1439	1446	O, MS, LRI	
5-Ethyl-2,4-dimethyl-thiazole	Popcorn, nutty, sweet, roasted	n.d.	n.d.	n.d.	1080	1425	1442	11	2	1080	1079	1425	1424	O, MS, LRI_S_	
2-Ethyl-4,5-dimethyl-3-thiazoline (I)	Roasted onion, garlic	5	1	1083	1094	n.d.	n.d.	10	2	1088	1085	n.d.	n.d.	MS, LRI_S_	
Tetramethylpyrazine	Cardboard, pyrazine, medicinal	n.d.	n.d.	n.d.	n.d.	n.d.	n.d.	26	5	1092	1088	n.d.	1458	O, MS, LRI	
5-Ethyl-2,4-dimethyl-3-thiazoline (II)	Fatty, beef fat, grilled meat, savoury	33	6	1095	1094	1441	1434	20	3	1098	1095	1453	1451	MS, LRI_S_	
Nonanal	Floral, fruity, fatty, citral, leafy	12	3	1103	1107	1387	1375	14	3	1108	1107	1396	1391	O, MS, LRI	
2-Acetyl-2-thiazoline	Toasted, popcorn	26	4	1109	1111	n.d.	n.d.	2	1	1110	n.d.	n.d.	n.d.	O, MS, LRI	
4-Methyl-2-isopropylthiazole	Roasting tin bits, pyrazine, seasoning, onions,	22	4	1116	1120	n.d.	n.d.	n.d.	n.d.	n.d.	n.d.	n.d.	n.d.	O, MS	
Unknown	Earthy, soil	-	-	-	-	-	-	16	3	1120	-	-	-	-	
2-Acetyl-3,4,5,6-tetrahydropyridine	Popcorn, rice cracker, roasted	n.d.	n.d.	n.d.	n.d.	n.d.	n.d.	20	4	1142	1142	1583	n.d.	O, ms, lri	[37]
(*Z*)-2-Nonenal	Fatty, sheets	24	4	1149	n.d.	1498	1489	n.d.	n.d.	n.d.	n.d.	n.d.	n.d.	O, ms, lri	[33,38]
(*E*,*E*)-2,6- Nonadienal	Violets, floral, waxy, fatty	29	6	1155	n.d.	1580	n.d.	n.d.	n.d.	n.d.	n.d.	n.d.	n.d.	O, LRI	
(*E*/*Z*)-3,5-Dimethyl-1,2,4-trithiolane	Fatty, roasted, (fried) rice	n.d.	n.d.	n.d.	n.d.	n.d.	n.d.	18	3	1158	1157	1582	1579	O, ms, lri	[35,39]
(*E*)-2-Nonenal	Fatty, waxy, aldehydic	29	6	1162	1165	1528	1529	15	3	1161	1165	1511	1524	O, MS, LRI	
2-Methyl-3-furyl methyl disulfide	Casserole, meaty, beefy, peppery	n.d.	n.d.	n.d.	n.d.	n.d.	n.d.	4	1	1185	1181	1630	1649	O, MS, LRI	
Decanal	Orange	n.d.	n.d.	n.d.	n.d.	n.d.	n.d.	6	1	n.d.	1209	1493	1490	O, MS, LRI	
2,4,6-Trimethyl-1,3,5-dithiazinane	Black onion, soil	n.d.	n.d.	n.d.	n.d.	n.d.	n.d.	17	4	1220	1221	1737	1736	O, ms, lri	[22]
Furfuryl methyl disulfide + (*E*,*E*)-2,4-Nonadienal	Chicken, fatty	n.d.	n.d.	n.d.	n.d.	n.d.	n.d.	5	1	1228	1226	n.d.	1799	O, MS, LRI	
		n.d.	n.d.	n.d.	n.d.	n.d.	n.d.			1228	n.d.	1699	1690	O, MS, LRI	
Benzothiazole	Savoury, chicken fat, nutty	n.d.	n.d.	n.d.	n.d.	n.d.	n.d.	18	4	1244	1249	1944	1937	O, MS, LRI	
(*E*/*Z*)-3-Ethyl-5-methyl-1,2,4-trithiolane	Blackcurrant, cat’s pee, crispy chicken skin	n.d.	n.d.	n.d.	n.d.	n.d.	n.d.	10	2	1262	1267	1699	1687	ms, lri	[40]
Nonanoic acid	Sweaty	n.d.	n.d.	n.d.	n.d.	n.d.	n.d.	9	2	1267	1268	n.d.	2179	O, MS, LRI	
(*E*,*E*)-2,4-Decadienal	McCains, fried, fatty, meaty	5	1	1337	1327	1812	1814	9	2	1331	1325	1814	1802	O, MS, LRI	
(*E*)-2-Undecenal	Herby, fatty	18	4	1372	1370	1715	n.d.	n.d.	n.d.	n.d.	n.d.	n.d.	n.d.	O, MS, LRI	
bis(2-Methyl-3-furyl) disulfide	Beef fat, meaty, fatty	5	1	1547	1550	n.d.	n.d.	n.d.	n.d.	n.d.	n.d.	n.d.	n.d.	O, MS, LRI	

^a^ Odour descriptors provided by 3 trained panellists; ^b^ Sum of odour intensities of duplicate samples recorded by panellists on DB-5 column (maximum score = 60); ^c^ Number of times the odour was detected by panellists (maximum *n* = 6); ^d^ Linear retention indices determined on DB-5 column (n.d. = not detected); ^e^ Linear retention indices determined on ZB-WAX column (n.d. = not detected); ^f^ Confirmation of identity where O = odour description agrees with literature; MS = mass spectrum agrees with that of authentic compounds; ms = mass spectrum agrees with reference spectrum in NIST 14/Inramass MS database or in literature; LRI = linear retention indices on DB-5 and/or ZB-WAX columns (where applicable) agree with that of authentic compounds; LRI_S_ = linear retention indices on DB-5 and/or ZB-WAX columns (where applicable) agree with that of compounds synthesised in the laboratory; lri = linear retention indices on DB-5 and/or ZB-WAX columns (where applicable) agree with literature values; ^g^ Literature reference for LRI values.

### 2.2. Differences between BB and BC

**Figure 1:**
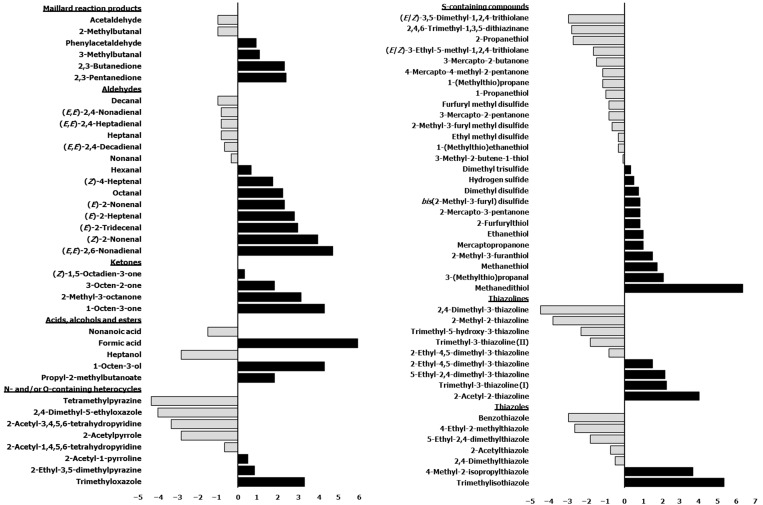
Difference in mean odour intensities of duplicate samples recorded by three panellists on DB-5 column between BB and BC extracts (positive values indicate that the compound was present at a stronger intensity in BB than BC and vice versa for the negative values).

Comparison of the two species showed that there were 21 volatiles identified in the BB extract which were not found in the BC extract and 14 volatiles vice versa. Although the majority of the volatiles were present in both the extracts, the odour intensities of these compounds varied, indicating their different levels of contribution to the aroma of each extract. Figure 1 depicts the difference in mean odour intensities between BB and BC, whereby positive values indicate that the compound was present at a stronger intensity in BB than BC and vice versa for the negative values. A few interesting observations could be made from this figure. The majority of the Maillard reaction products, such as the Strecker aldehydes and diketones, occurred at higher intensities in BB than BC. Meanwhile, among the lipid-derived aldehydes, the alkadienals were more predominant in BC while alkenals were more prevalent in BB.

Some fatty odorants, such as (*E*,*E*)-2,4-heptadienal and (*E*,*E*)-2,4-nonadienal, were only found in the BC extract, while others such as (*E*)-2-heptenal (green, citral, waxy), (*Z*)-2-nonenal (fatty, plastic-like), (*E*,*Z*)-2,6-nonadienal (violets, floral, waxy, fatty) were only found in the BB extract. However, the strength of fatty odours in the overall aroma is not a consequence of the quantity of identified aldehydes, and the odour activities of the volatiles ought to be considered (Table 2). Thus, it was possible that fatty odorants had a greater contribution to BC than BB aroma, as reported by Gasser and Grosch [11] who observed a stronger prevalence of fatty odorants such as (*E*,*E*)-2,4-decadienal, γ-dodecalactone and (*E*/*Z*)-2-undecenal in the aroma of BC as compared to BB as a result of the higher fat content in the former. Although (*E*,*E*)-2,4-decadienal was present in both extracts, the total odour intensity was almost twice as strong in the BC extract as compared to the BB extract.

As for the Strecker degradation products, 3-methylbutanal, phenylacetaldehyde and 3-(methylthio)-propanal were present at higher intensities in BB than BC as seen in Figure 1. More notably, bis(2-methyl-3-furyl) disulfide (beef fat, meaty, fatty) were only found in the BB extract. This latter compound, with an extremely low odour threshold value of 2.4 × 10^−5^ μg kg^−1^ in water [44], is a dimer of 2-methyl-3-furanthiol [45] and was also found in several other studies [7,46,47]. These results corroborated the findings of Gasser and Grosch [11] that bis(2-methyl-3-furyl) disulfide and the Strecker aldehydes were more predominant in the aroma of boiled beef. Furthermore, these authors also reported 12-methyltridecanal to be a beef-specific odorant with tallowy, beef-like notes, which was proposed to be liberated from plasmalogens in ruminants after a long heating period [48,49]. However, this branched aldehyde was not found in our study, which could be attributed to the milder heating conditions and shorter duration impeding the release of the aldehyde from plasmalogens, as it was also not reported in several other studies with similar experimental conditions [8,11,50,51,52].

### 2.3. 3-Thiazolines and Thiazoles 

**Figure 2:**
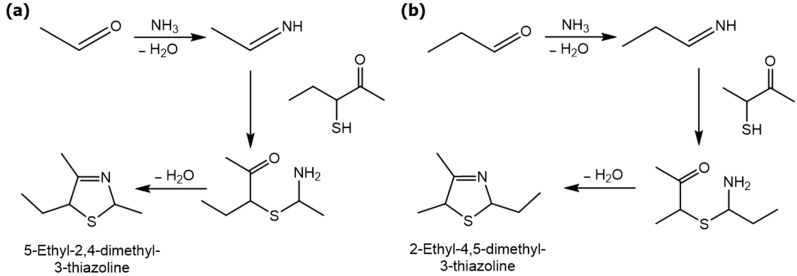
Proposed scheme for the formation of (**a**) 5-ethyl-2,4-dimethyl-3-thiazoline and (**b**) 2-ethyl-4,5-dimethyl-3-thiazoline.

**Figure 3:**
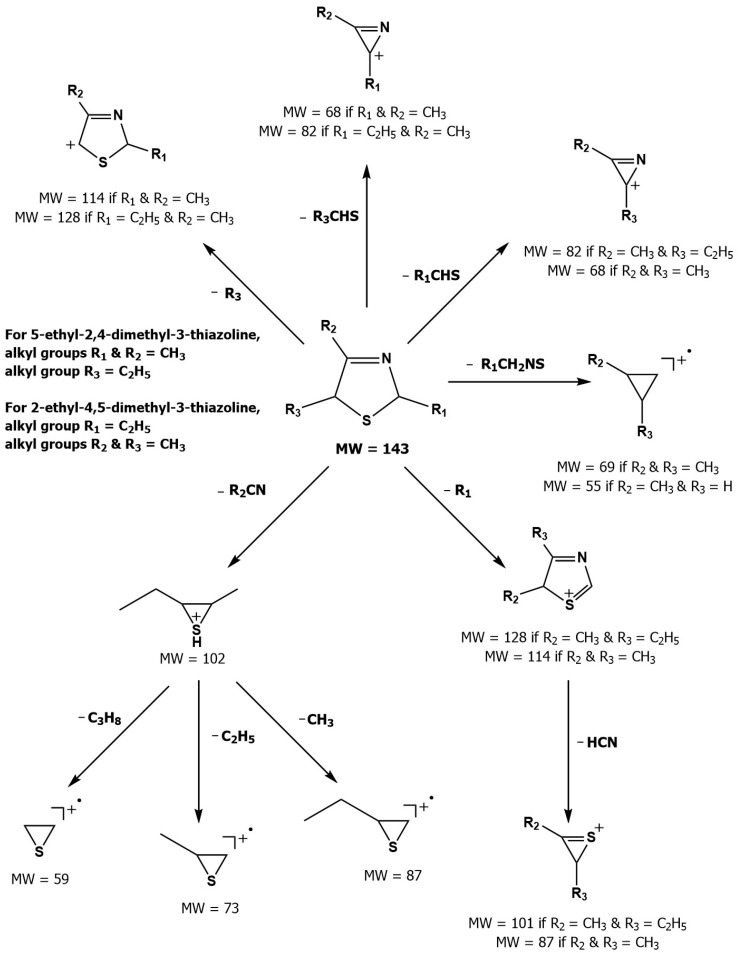
Mass fragmentation pattern of 5-ethyl-2,4-dimethyl-3-thiazoline and 2-ethyl-4,5-dimethyl-3-thiazoline (Adapted with permission from Elmore et al. [53] and Bredie et al. [54]. Copyright 1997 and 2002 American Chemical Society).

For the first time, 5-ethyl-2,4-dimethyl-3-thiazoline (fatty, grilled meat, savoury) and 2-ethyl-4,5-dimethyl-3-thiazoline (roasted onion, garlic) were reported in BB and BC extracts. Not only are these 3-thiazolines structural isomers, but each also exists as geometric isomers represented by distinct peaks of similar ratios (based on percentage of total ion chromatogram peak area). The mass spectra of these isomers are provided in Table 3 while their formation mechanisms and mass fragmentation patterns are proposed in Figure 2 and Figure 3, respectively.

The formation pathway was proposed to be the reaction of α-hydroxyketones or α-dicarbonyls and aliphatic aldehydes in the presence of hydrogen sulfide and ammonia, yielding 3-thiazolines which form thiazoles upon oxidation [53]. The nucleophilic addition of -SH to the carbonyl group of α-dicarbonyls, followed by reduction, resulted in the formation of mercaptoketones, which could react with the imine intermediate, formed between ammonia and the aldehyde, in a nucleophilic attack. The subsequent ring closure and water elimination resulted in the formation of 3-thiazolines. Thus, in addition to the α-hydroxyketones and α-dicarbonyls, the presence of mercaptoketones, which are intermediates in the mechanism, could also result in the formation of 3-thiazolines and thiazoles. Since these precursors were identified in the GC-MS and GC-O, the formation of 3-thiazolines and thiazoles in the BB and BC extracts is very likely. Given the high odour intensity scores of these compounds, they could also be important contributors to boiled meat aromas.

Other 3-thiazolines and several thiazoles were also identified in this study, among which 2,4-dimethyl-3-thiazoline (meaty, brothy, roasted), 2,4-dimethylthiazole (meaty, grilled chicken, roasted), trimethyl-3-thiazoline (pickled onion, cat’s pee; meaty, fried onion), and 5-ethyl-2,4-dimethylthiazole (popcorn, nutty, sweet and roasted) had been found in beef with odour thresholds of 0.02, 0.5, 0.1 and 0.002 mg × kg^−1^ in water, respectively [55]. The last two compounds were also found in fried chicken, along with 4-ethyl-2-methylthiazole (popcorn, basmati, biscuit, roasting skin) [56]. These authors also suggested that the lack of mention of 3-thiazolines in literature is due to their presence in trace quantities and susceptibility to oxidation. The presence of thiazolines also exists in other foods such as 2-ethyl-4,5-dimethyl-3-thiazoline in freeze-dried onion sprout [57], as well as trimethylisothiazole (odd sulfur, cardboard, earthy, green) in yeast extract paste [35] and sesame seed oil [58].

Among all the 3-thiazolines and thiazoles with confirmed identities (i.e., by odour description, GC-O and GC-MS LRIs), 2,4-dimethyl-3-thiazoline and 4-ethyl-2-methylthiazole were only identified in BC. These compounds share a common roast aroma, with the thiazoline possessing a meaty note and the thiazole bearing sweeter or more fragrant attributes such as popcorn, basmati and barley. The toasted and rice qualities could also be enhanced in BC by the higher odour intensity score of 2-acetylthiazole in BC than BB (24 vs. 20). Meanwhile, the 3-thiazolines present in BB were characterised by stronger savoury (onion and meaty) notes as manifested in the higher odour intensity scores for 5-ethyl-2,4-dimethyl-3-thiazoline (33 in BB vs. 20 in BC) and trimethyl-3-thiazoline isomer I (31 in BB vs. 17 in BC). Overall, 3-thiazolines and thiazoles contributed to the toast, nutty and rice notes in the BC aroma to create a more rounded profile, while the onion and meaty attributes could be enhanced in the BB aroma to produce a more intense savoury perception.

## 3. Materials and Methods

### 3.1. Reagents and Chemicals 

Aroma chemicals were obtained from the following suppliers and were ≥95% in purity unless stated otherwise: acetaldehyde, hexanal, heptanal, octanal, nonanal, decanal, 3-(methylthio)propanal, 2-methylbutanal, (*E*)-2-nonenal, (*E*)-2-undecenal, (*E*,*E*)-2,4-heptadienal (90%), (*E*,*E*)-2,4,-nonadienal (≥89%), (*E*,*E*)-2,6-nonadienal, 1-heptanol, 1-octen-3-ol, (*Z*)-4-heptenal, 1-octen-3-one, 2,3-butanedione, 2,3-pentanedione, 3-mercapto-2-butanone, dimethyl disulfide, dimethyl trisulfide, benzothiazole, 2-methyl-3-furyl methyl disulfide, 2-acetylpyrrole, 2-acetyl-2-thiazoline, tetramethylpyrazine and 2-isopropylpyrazine were from Sigma Aldrich (Gillingham, UK); 4-nonanone, 2-furylmethanethiol, 3-mercapto-2-pentanone, 2-methyl-2-thiazoline and 1-(methylthio)propane from TCI (Oxford, UK); (*E*,*E*)-2,4-decadienal (90%) and trimethyloxazole from Lancaster Synthesis (Heysham, UK); phenylacetaldehyde and 3-octen-2-one from Acros Organics (New Jersey, USA); 1-(methylthio)ethanethiol from Carbosynth (Newbury, UK); 1-propanethiol from Fisher Scientific (Loughborough, UK); furfuryl methyl disulfide, 2,4-dimethylthiazole and (*E*)-2-heptenal from Oxford Organics; 2-acetylthiazole from Riverside Aromatics (Poole, UK); 2-ethyl-3,5-dimethylpyrazine from Fluorochem (Hadfield, UK); 3-methylbutanal from Alfa Aeser (Heysham, UK); 2-methyl-3-furanthiol from IFF (Haverhill, UK), 3-methyl-2-butene-1-thiol from AROXA (Surrey, UK), 2-acetyl-1-pyrroline (≥85%) from aromaLAB GmbH (Martinsried, Germany), nonanoic acid from Anitox (Wellingborough, UK). Pentane (≥98%), diethyl ether (≥99.5%), dichloromethane (≥99.8%), ammonium sulfide (20% wt. solution in H_2_O), propanal (97%) and 1-hydroxy-2-butanone (Aldrich^CPR^; purity unknown) were from Sigma-Aldrich. 1-Hydroxy-2-propanone (97%) was from Fluka (Seelze, Germany). High purity water (18.2 MΩ) was obtained from a Select Fusion Ultrapure water deionisation unit (SUEZ, Peterborough, UK).

### 3.2. Likens-Nickerson Simultaneous Distillation-Extraction (LN-SDE) 

Fresh beef silverside joint and fresh Class A chicken breast were purchased from a retail supermarket and used within the sell-by date. The meats were provided by one commercial supplier and from the same batch and farm. The chickens were a standard Ross 308 genotype and were reared, slaughtered and processed under commercial conditions. The meat was trimmed of extramuscular fat and minced using a food mincer with 4.5 mm screen (Kenwood, Hampshire, UK). The minced meat (500 g) and high purity water (500 g) were added to a round bottom flask. The sample was boiled at 100 °C in a heating mantle for 30 min. Likens-Nickerson SDE was performed using 30 mL of redistilled pentane/diethyl ether (9:1 *v/v*) for 2 h. The extract was concentrated to 0.5 mL using a Vigreux column and stored at −80 °C prior to analysis.

### 3.3. Gas-Chromatography Olfactometry (GC-O)

GC-O analyses were performed on a HP 5890 Series II GC equipped with a flame ionisation detector (Hewlett Packard, Waldbronn, BaWü, Germany) and an ODO II odour port (SGE, Ringwood, Victoria, Australia). The instrument was controlled with ChemStation (version A.06.03). An aliquot of sample (2 µL) was injected in splitless mode, with the injector at 250 °C. Chromatographic separation was achieved on two columns of different polarities. For the non-polar Rxi-5 Sil MS column (30 m × 0.25 mm × 1 μm; Restek, Bellefonte, PA, USA), the oven temperature was programmed from 35 °C to 200 °C at 6 °C min^−1^ and finally to 320 °C at 15 °C min^−1^. For the polar ZB-Wax column (30 m × 0.25 mm × 0.25 μm; Phenomenex, Torrance, CA, USA), the oven temperature was programmed from 35 °C to 200 °C at 6 °C min^−1^ and finally to 250 °C at 15 °C min^−1^. All initial and final temperatures were held for 10 min. Helium was used as the carrier gas at a constant flow rate of 2.0 mL min^−1^. The column effluent was split equally between the FID and odour port, where the odours of the eluting components were evaluated. The samples were analysed in duplicates by 3 trained assessors who were screened for olfactory performance in terms of threshold, discrimination and identification using a Sniffin’ Sticks test (Burghardt^®^, Wedel, Germany). Each has an olfactory score of ≥38 (out of a maximum of 48) and at least one year of GC-O experience. Assessors recorded the descriptions and intensity scores on a scale of 1–10 (where 3 = weak, 5 = medium and 7 = strong) for the odours detected. 

### 3.4. Gas Chromatography-Mass Spectrometry (GC-MS) 

GC-MS analyses were performed on an Agilent 7890A GC coupled to an Agilent 5975C inert XL EI/CI MSD triple axis MS (Agilent Technologies, Santa Clara, CA, USA) and controlled with Agilent MSD ChemStation (version E.02.02). An aliquot of sample (2 µL) was injected in splitless mode, with the injector at 250 °C. Chromatographic separation was achieved on two columns of different polarities. For the non-polar HP-5 MS column (30 m × 0.25 mm × 1 μm; Agilent Technologies), the oven temperature was increased from 35 °C to 320 °C at 6 °C min^−1^. For the polar ZB-Wax column (60 m × 0.25 mm × 0.25 μm; Phenomenex), the oven temperature was increased from 35 °C to 250 °C at 4 °C × min^−1^. All initial and final temperatures were held for 10 min. The carrier gas was helium at a constant pressure of 124 kPa. The MS was operated in electron impact mode with a source temperature of 250 °C, ionisation energy of 70 eV and a scan range from *m/z* 29 to *m/z* 400.

### 3.5. Compound Identification and Quantification 

The chromatograms were processed using Agilent MSD ChemStation (version F.01.03.2365; Agilent Technologies). A series of C_5_–C_25_ n-alkanes was analysed under the same conditions for the calculation of the linear retention index (LRI) of each compound. The identities of the compounds were confirmed based on a match of their mass spectra, LRI and odour descriptors with those of authentic compounds where available. Otherwise, a tentative identification was made by comparing their mass spectrum against the NIST 14 NIST/EPA/NIH EI MS Library (Gaithersburg, MD, USA) or INRAMASS MS library (INRA, France), as well as LRI and odour descriptors available in the literature.

### 3.6. 3-Thiazoline and Thiazole Synthesis 

Synthesis was carried out using an adapted method from Elmore and Mottram [53]. Equimolar quantities (5 mmol) of an aldehyde and a mercaptoketone or α-hydroxyketone depending on availability (Table 4) were added to 50 mL of 0.1 M ammonium sulfide solution in a Duran bottle before heating in a 100 °C water bath for 30 min with constant stirring using a magnetic stirrer. The reaction mixtures were allowed to cool at room temperature before extraction with 30 mL dichloromethane. The extracts were flushed under nitrogen before storage at −20 °C until analysis by GC-MS as described in Section 3.4.

## 4. Conclusions

This study has demonstrated the important contribution of 3-thiazolines in boiled meat aroma and their high aroma intensities could alter and/or enhance the meaty profile in BB and BC. The identification of 5-ethyl-2,4-dimethyl-3-thiazoline and 2-ethyl-4,5-dimethyl-3-thiazoline was also reported in BB and BC for the first time. Aroma extract dilution analysis and recombination studies could be carried out to further evaluate their importance. Nevertheless, the trends were that S-containing compounds and Strecker aldehydes were more prevalent in BB than BC, while fatty odorants, which were mainly the lipid-derived aldehydes, were more predominant in BC than BB. This information would be useful for the creation of species-specific meat flavourings or those directed towards desired profiles.

## Figures and Tables

**Table 2:** Odour thresholds of selected alkenals and alkadienals.

Alkenal/Alkadienal	Odour Threshold (μg kg^−1^ in Water)	Source
(*E*)-2-Heptenal	13	[41]
(*E*,*E*)-2,4-Heptadienal	0.032	[42]
(*E*,*E*)-2,4-Nonadienal	0.06	[43]
(*E*,*Z*)-2,6-Nonadienal	0.0045–0.02	[42,43]
(*E*,*E*)-2,4-Decadienal	0.027–0.07	[41,42]

**Table 3:** Mass spectral data for 5-ethyl-2,4-dimethyl-3-thiazoline and 2-ethyl-4,5-dimethyl-3-thiazoline.

Compound	LRI_DB-5_^a^	LRI_ZB-Wax_^b^	Mass Spectral data, *m/z* (Relative Intensity) ^c^
5-Ethyl-2,4-dimethyl-3-thiazoline (isomer I)	1080	1419	69, 42 (72), 102 (64), 73 (44), **143** (43), 41 (38), 68 (36), 45 (33), 60 (32), 114 (27)
5-Ethyl-2,4-dimethyl-3-thiazoline (isomer II)^†^	1099	1446	69, 42 (70), 102 (58), 73 (40), **143** (40), 41 (39), 68 (34), 45 (34), 60 (30), 114 (25)
2-Ethyl-4,5-dimethyl-3-thiazoline (isomer I)^†^	1089	1443	114, 68 (67), 102 (44), **143** (55), 87 (27), 69 (20), 42 (20), 60 (20), 55 (15), 56 (14)
2-Ethyl-4,5-dimethyl-3-thiazoline (isomer II)	1100	1465	114, 68 (68), 102 (47), **143** (40), 87 (35), 42 (35), 69 (30), 45 (25), 41 (23), 39 (23)

† Compound identified in boiled beef and boiled chicken aroma extracts; ^a^ Linear retention index on DB-5 column; ^b^ Linear retention index on ZB-Wax column; ^c^ First number is the base peak; molecular ion in bold type.

**Table 4:** Precursors for 3-thiazoline and thiazole synthesis.

Reactants		Products	
Aldehyde	α-Hydroxyketone/ Mercaptoketone	3-Thiazoline	Thiazole
**Acetaldehyde**	1-Hydroxy-2-propanone	2,4-Dimethyl-	
		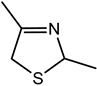	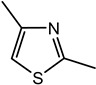
**Acetaldehyde**	1-Hydroxy-2-butanone	4-Ethyl-2-methyl-	
		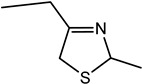	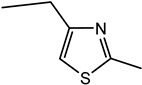
**Acetaldehyde**	3-Mercapto-2-pentanone	5-Ethyl-2,4-dimethyl-	
		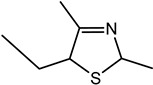	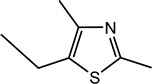
**Propanal**	3-Mercapto-2-butanone	2-Ethyl-4,5-dimethyl-	
		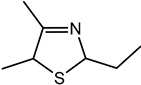	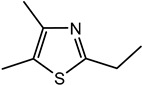
**Acetaldehyde**	3-Mercapto-2-butanone	Trimethyl-	
		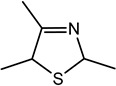	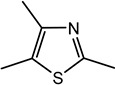

## Data Availability

All data are included in the article.

## Sample Availability

Samples are not available due to sample instability and short shelf life.
